# Decreased rates of advanced breast cancer due to mammography screening in The Netherlands

**DOI:** 10.1038/sj.bjc.6602075

**Published:** 2004-08-03

**Authors:** J Fracheboud, S J Otto, J A A M van Dijck, M J M Broeders, A L M Verbeek, H J de Koning, and National Evaluation Team for Breast cancer screening (NETB)

**Affiliations:** 1 Department of Public Health, Erasmus MC, University Medical Center Rotterdam, PO Box 1753, NL-3000 DR Rotterdam, The Netherlands; 2 Netherlands Cancer Registry, Association of Comprehensive Cancer Centres, PO Box 19001, NL-3501 DA Utrecht, The Netherlands; 3 Comprehensive Cancer Centre East, PO Box 1281, NL-6501 BG Nijmegen, The Netherlands; 4 Department of Epidemiology and Biostatistics, University Medical Centre Nijmegen, PO Box 9101, 6500 HB Nijmegen, The Netherlands

**Keywords:** breast cancer incidence, advanced breast cancers, breast cancer screening, breast cancer mortality

## Abstract

The effect of the implementation of the Dutch breast cancer screening programme during 1990–1997 on the incidence rates of breast cancer, particularly advanced breast cancer, was analysed according to stage at diagnosis in seven regions, where no screening took place before 1990. The Netherlands Cancer Registry provided detailed data on breast cancer incidence in 1989–1997 by tumour stage, age and region. Annual age-adjusted incidence rates of all breast cancers and advanced cancers, defined as large tumours T2+ with lymph node and/or distant metastases, were compared with rates in 1989. In general, breast cancer incidence rose strongly in the early 1990s, especially in the age category 50–69 years (estimated annual percentage change (EAPC) 4.25; 95% CI 1.70, 6.86). The increase was mainly due to the increase in small T1 cancers and ductal carcinoma *in situ*. However, in women aged 50–69, advanced cancer incidence rates showed a significant decline by 12.1% in 1997 compared with 1989 (EAPC –2.14, 95% CI −3.47, −0.80), followed by a breast cancer mortality reduction of similar size after approximately 2 years. We confirm that breast cancer screening initially leads to a temporary strong increase in the breast cancer incidence, which is followed by a significant decrease in advanced diseases in the women invited for screening. It is evident that breast cancer screening contributes to a reduction in advanced breast cancers and breast cancer mortality.

Large-scale early detection of breast cancer leads to an increase in newly diagnosed cases and a shift of breast cancer stages towards higher proportions of less severe tumours. The effects of breast cancer screening on the overall breast cancer incidence depend on the characteristics of the screening programme such as the targeted age range, the duration of implementation, the extent of coverage and attendance, the screening interval and the programme performance. A decrease in advanced disease stages is an important predictor of the potential breast cancer mortality reduction by mammography screening (Day *et al*, 1989).

Population-based mammography screening started in the mid-1970s in two Dutch regions in and around the cities of Utrecht and Nijmegen (‘old’ regions). From 1989 to 1997, nation-wide breast cancer screening was implemented in The Netherlands for all women aged 50–69 years. Early outcomes of the nation-wide programme, such as attendance rate, breast cancer detection and tumour stage distribution of screen-detected breast cancers, were largely in line with the expectations that were based on a cost-effectiveness analysis (de Koning *et al*, 1995; Fracheboud *et al*, 2001). During the 1990s, Netherlands Cancer Registry (NCR) data showed increases in breast cancer incidence and changes in stage distribution, particularly in the age category targeted for screening (van Dijck *et al*, 2000). However, data from the registry cannot distinguish screened from nonscreened women.

In this study, we describe stage-specific trends in the breast cancer incidence 1989–1997 during the implementation of the nation-wide breast cancer screening programme in The Netherlands. As we observed a significant decrease in breast cancer mortality within the population targeted for screening as of 1997 (Otto *et al*, 2003), we assumed a decline in advanced disease stages as well. For this reason, we focused our analyses on trends in advanced tumour stages in the seven regions that started screening activities after 1990 (‘new’ regions).

## MATERIAL AND METHODS

### Setting

The nation-wide breast cancer screening programme for women aged 50–69 years was gradually implemented in The Netherlands during 1989–1997. Every 2 years, eligible women get a personal invitation letter with a fixed appointment for a screen examination in one of the mostly mobile screening units. Over 75% of the invited women attend the programme and more than 90% of the attendees reattend in the following round. Experimental, nonrandomised breast cancer screening started in the mid-1970s in the city of Utrecht (central region) and its environs, and in the city of Nijmegen (eastern region). The pilot programmes were gradually integrated in the national programme around 1989. In 1990, three other regions started screening activities, followed by the remaining four regions in 1991. Up to 1994, the majority of screen examinations were initial screens, but since 1995 subsequent screens have been carried out predominantly. The number of screen examinations increased from 1300 per 100 000 women aged 50–69 in 1990 to more than 35 000 per 100 000 in 1997.

The first regional cancer registry was established in the 1950s in the southeast of The Netherlands. Only in the 1980s, cancer registries were launched in the other eight regions. In 1989, coverage was nation-wide, so that the regional registries could be brought together to a national database, the Netherlands Cancer Registry (NCR, 1992). Cancer recording is based on notifications of malign pathology by the computerised national histopathological database (PALGA). The records are complemented with clinical information by the regional Comprehensive Cancer Centres and checked for missing cases by comparing them with the national registry of outpatient and in-patient diagnoses (LMR). The completeness of case ascertainment of the NCR is higher than 95% (Visser *et al*, 2003); this percentage will be still higher for breast cancer, because histological or cytological material is available for nearly all cases. Tumour characteristics are registered according to the international guidelines for classification of stage (TNM, International Union Against Cancer (UICC)) and topography and morphology (International Classification of Diseases for Oncology (ICD-O)). The classification is primarily based on histopathological TNM information; in case of unknown pN (=X) clinical information cN is used. Usually, it takes 3 years to complete and publish the data on the cancer incidence of a certain year.

### Method

The National Evaluation Team for Breast cancer screening in The Netherlands (NETB) annually collects regional aggregated data on the screening results, including attendance, referral recommendations, screen-detected breast cancers and stage distribution. Files of regional screening records are linked to the corresponding regional cancer registry database to identify interval cancers. These data are also provided to the NETB, which analyses trends in incidence and therapy of screen-detected cancers, interval cancers and breast cancers in nonscreened women.

In 2001, the NCR provided data on population, breast cancer incidence, tumour size, positive or negative lymph node stage and distant metastases by calendar year 1989–1997 and 5-year age groups for the nine cancer registry regions separately. These regions largely correspond to the nine breast cancer screening regions in The Netherlands. The data include ductal carcinoma *in situ*, but not lobular carcinoma *in situ*, which is regarded a benign lesion. To be able to study the effect of introducing a screening programme in a previously unscreened population, the NCR data were subdivided into two ‘old’ regions (the central and eastern regions where the pilot programmes took place) and into seven ‘new’ regions that did not start screening activities until 1990.

For the national evaluation, the age at screening and of breast cancer incidence is defined by birth year; that is, during a certain year, the woman is considered to keep the age she had on January 1 of the same year. For this reason, women will be invited and screened for the first time in the year when they become 50 years old, thus having the evaluation age of 49.

As we did not have detailed information on the lymph node status, we did not use UICC TNM stages, but grouped invasive breast cancers into six categories: (1) small tumours T1 (up to 20 mm in size) without metastases (T1N0); (2) small tumours T1 with lymph node or distant metastases (T1N+/M1); (3) small tumours T1 with unknown lymph node status (T1Nx); (4) large cancers T2+ (T2: 20–50 mm in size, T3: >50 mm in size and T4) without metastases (T2+N0); (5) large cancers T2+ with lymph node or distant metastases (T2+N+/M1) and (6) large cancers T2+ with unknown lymph node status (T2+Nx). We defined category (5) T2+N+/M1 as ‘advanced cancers’ in our study. The proportion of Tx and unclassified cancers varied between 2.1 and 3.2% of the annual breast cancer total. The same variation was observed within 5-year age groups, except the oldest age group (>79 years) in which the proportion of Tx and unclassified cancers was approximately 5%.

All breast cancer incidence rates were calculated per 100 000 women by dividing the number of new breast cancer cases in a certain year by the mid-year female population (the average of the population at January 1 of that year and the population at January 1 of the following year). As the NCR did not supply population figures of January 1, 1998, we estimated it for the individual cancer registry regions by multiplying the 1997 regional age-specific population numbers by the age-specific percent change between 1997 and 1998 of the national population. All rates were age-adjusted by means of direct standardisation using the European Standard Population as reference.

Assuming a constant change of incidence rates over time, we estimated trends using the estimated annual percentage change (EAPC) by fitting a regression line to the natural logarithm of the incidence rates as dependent variable, and testing the slope by the *t*-distribution (number of degree of freedom equals the number of calendar year minus 2). The significance level of differences in advanced cancer incidence rates was estimated by means of the expected standard deviation of the difference in incidence rate of a certain year and age group compared with 1989, according to the method that we used for assessing changes in breast cancer mortality (van den Akker-van Marle *et al*, 1999; Otto *et al*, 2003).

## RESULTS

Table 1

**Table 1:** Annual age-adjusted invasive and ductal *in situ* breast cancer rates (ESR per 100 000 woman-years) in 1989–1997 and per cent change since 1989

	**Seven ‘new’ regions^a^**			**Two ‘old’ regions^b^**			**Netherlands (nine regions)**		
	**Breast cancer**		**Change (1989=0)**	**Breast cancer**		**Change (1989=0)**	**Breast cancer**		**Change (1989=0)**
**Age category**	***N***	**Rate**	**%**	***N***	**Rate**	**%**	***N***	**Rate**	**%**
<*50 years*									
1989	1826	43.2	—	372	42.7	—	2198	43.1	—
1990	1930	44.8	3.6	386	43.8	2.5	2316	44.6	3.4
1991	2018	45.6	5.6	455	50.2	17.6	2473	46.4	7.6
1992	2057	45.3	4.8	452	48.7	13.9	2509	45.9	6.3
1993	2201	47.5	9.9	442	46.2	8.0	2643	47.2	9.6
1994	2278	48.3	11.7	473	48.3	12.9	2751	48.3	11.9
1995	2267	47.2	9.2	444	44.6	4.3	2711	46.7	8.4
1996	2375	49.2	14.0	516	51.5	20.4	2891	49.6	15.1
1997	2376	49.6	14.8	509	51.1	19.6	2885	49.9	15.6
EAPC (95% CI)		1.63 (1.47, 2.09)				1.59 (−0.18, 3.39)		1.62 (1.05, 2.2)	
									
*50–69 years*									
1989	2795	225.8	—	671	284.2	—	3466	235.3	—
1990	2999	240.8	6.6	726	301.8	6.2	3725	250.7	6.5
1991	3316	265.6	17.6	743	304.3	7.1	4059	271.9	15.5
1992	3821	304.3	34.8	820	338.3	19.0	4641	309.9	31.7
1993	4059	323.6	43.3	846	347.3	22.2	4905	327.5	39.2
1994	4105	325.0	43.9	810	323.7	13.9	4915	324.8	38.0
1995	3968	312.0	38.2	746	296.4	4.3	4714	309.4	31.5
1996	4082	314.5	39.3	704	273.7	−3.7	4786	307.8	30.8
1997	4160	313.0	38.6	797	300.4	5.7	4957	310.9	32.1
EAPC (95% CI)		4.25 (1.70, 6.86)				−0.28 (−2.77, 2.28)		3.45 (0.98, 5.99)	
									
>*69 years*									
1989	2004	211.2	—	344	210.0	—	2348	211.0	—
1990	2075	208.4	−1.3	458	249.2	18.7	2533	214.8	1.8
1991	2123	210.6	−0.3	425	238.3	13.5	2548	214.9	1.9
1992	2268	221.5	4.9	465	235.8	12.3	2733	223.8	6.1
1993	2259	213.2	1.0	416	197.0	−6.2	2675	210.7	−0.2
1994	2583	240.2	13.8	453	221.8	5.6	3036	237.3	12.5
1995	2400	223.0	5.6	470	221.3	5.4	2870	222.7	5.6
1996	2489	221.0	4.6	472	228.9	9.0	2961	222.3	5.3
1997	2490	219.8	4.1	463	215.6	2.7	2953	219.1	3.9
EAPC (95% CI)		0.89 (−0.29, 2.09)				−0.60 (−2.82, 1.68)		0.64 (−0.43, 1.72)	
									
*All ages*									
1989	6625	101.3	—	1387	112.4	—	8012	103.1	—
1990	7004	106.1	4.8	1570	122.8	9.2	8574	108.8	5.5
1991	7457	112.3	10.9	1623	125.5	11.6	9080	114.5	11.1
1992	8146	121.5	20.0	1737	133.6	18.8	9883	123.5	19.8
1993	8519	126.7	25.1	1704	130.3	15.9	10 223	127.3	23.5
1994	8966	130.2	28.6	1736	127.9	13.8	10 702	129.8	25.9
1995	8635	124.7	23.2	1660	120.2	6.9	10 295	124.0	20.2
1996	8946	127.1	25.5	1692	119.4	6.2	10 638	125.8	22.1
1997	9026	126.7	25.1	1769	124.4	10.6	10 795	126.3	22.5
EAPC (95% CI)		2.90 (1.40, 4.43)				0.32 (−1.33, 2.00)		2.46 (0.98, 3.96)	

a Started screening activities in 1990 or 1991.

b Screening activities before 1989 (regions with pilot programmes).

EAPC=estimated annual percentage change.

gives the age-adjusted combined invasive and *in situ* breast cancer incidence rates in 1989–1997 and the percent change compared with 1989 by different age categories. In the total Dutch female population, incidence rates increased in all age groups. They reached a maximum in 1993–1994 and then slightly declined but still remained higher than the initial level. The overall incidence was highest in 1994 (25.9% increase compared to 1989). In women aged 50–69, the incidence rate reached a maximum in 1993 (39.2% increase compared to 1989). In younger women, the increase was more gradual and reached its maximum in 1996–1997 (+15.6% compared with 1989), whereas in older women the increase did not exceed 12.5%.

The increase of incidence rates was more pronounced in the ‘new’ regions, especially in women aged 50–69, in whom the incidence rate remained beyond 300 per 100 000 from 1992 onwards (EAPC 4.25; 95% CI 1.70, 6.86; Table 1). These rates are approx. 40% higher than the incidence rate in 1989 (225.8 per 100 000), which can be regarded as baseline incidence in an unscreened population in The Netherlands. In the ‘old’ regions, in women aged 50–69, the breast cancer incidence was substantially higher in 1989 (284.2 per 100 000) and reached the highest incidence level in 1993 with 347.3 per 100 000 (increase by 22.2%). However, there was no significant change of incidence rates over the total period 1989–1997.

Figure 1

**Figure 1:**
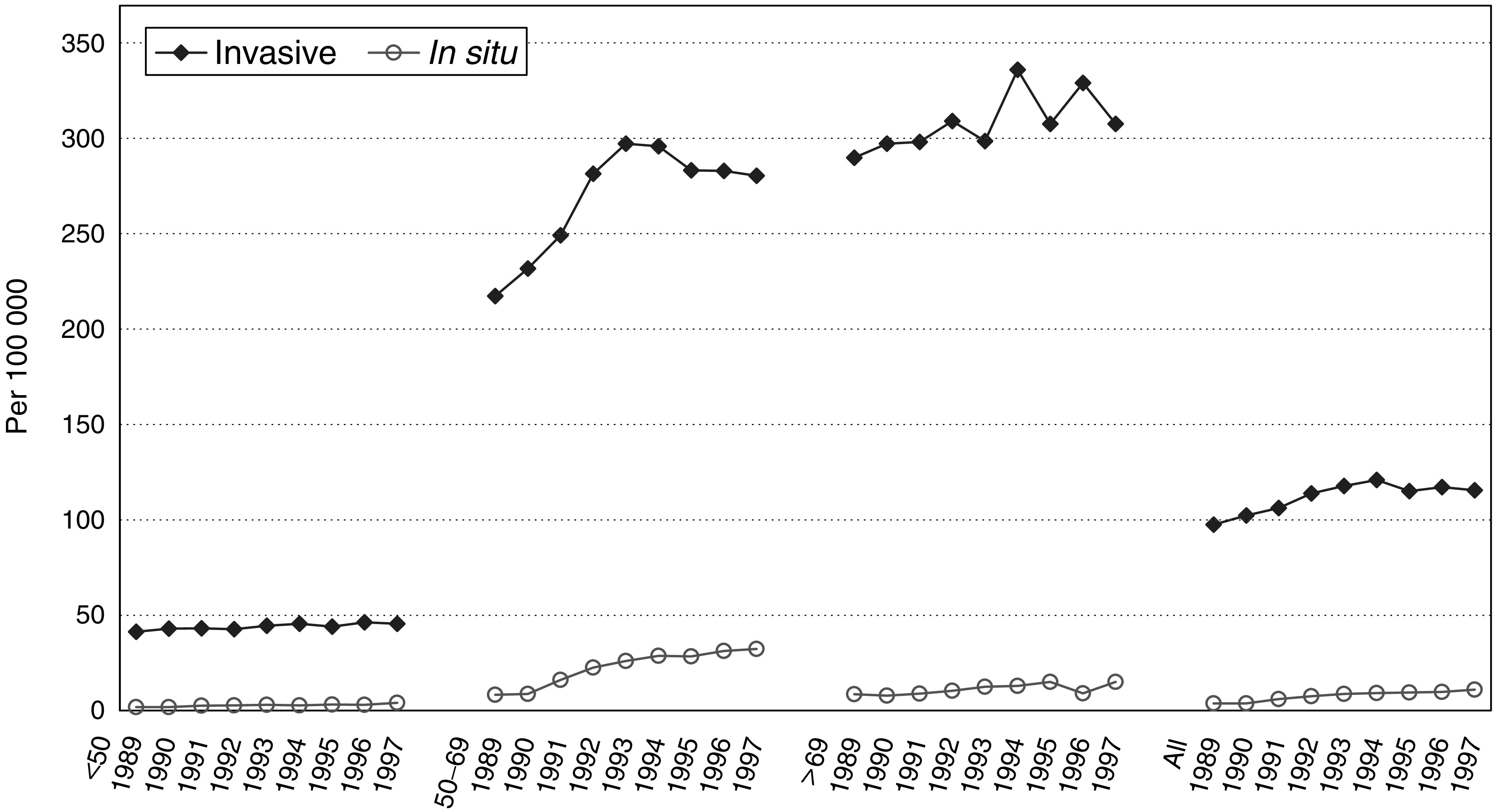
Age-adjusted invasive and *in situ* breast cancer incidence per 100 000 by age category, seven ‘new’ regions 1989–1997.

shows that the incidence rates of both, invasive and *in situ* breast cancers, increased in all age categories in the seven ‘new’ regions, but that this increase was the strongest in women aged 50–69. In 1997, a quarter of all newly diagnosed invasive and *in situ* breast cancers were detected by the screening programme, and one out of eight was diagnosed in screened women during the interval period between two screening rounds (Figure 2

**Figure 2:**
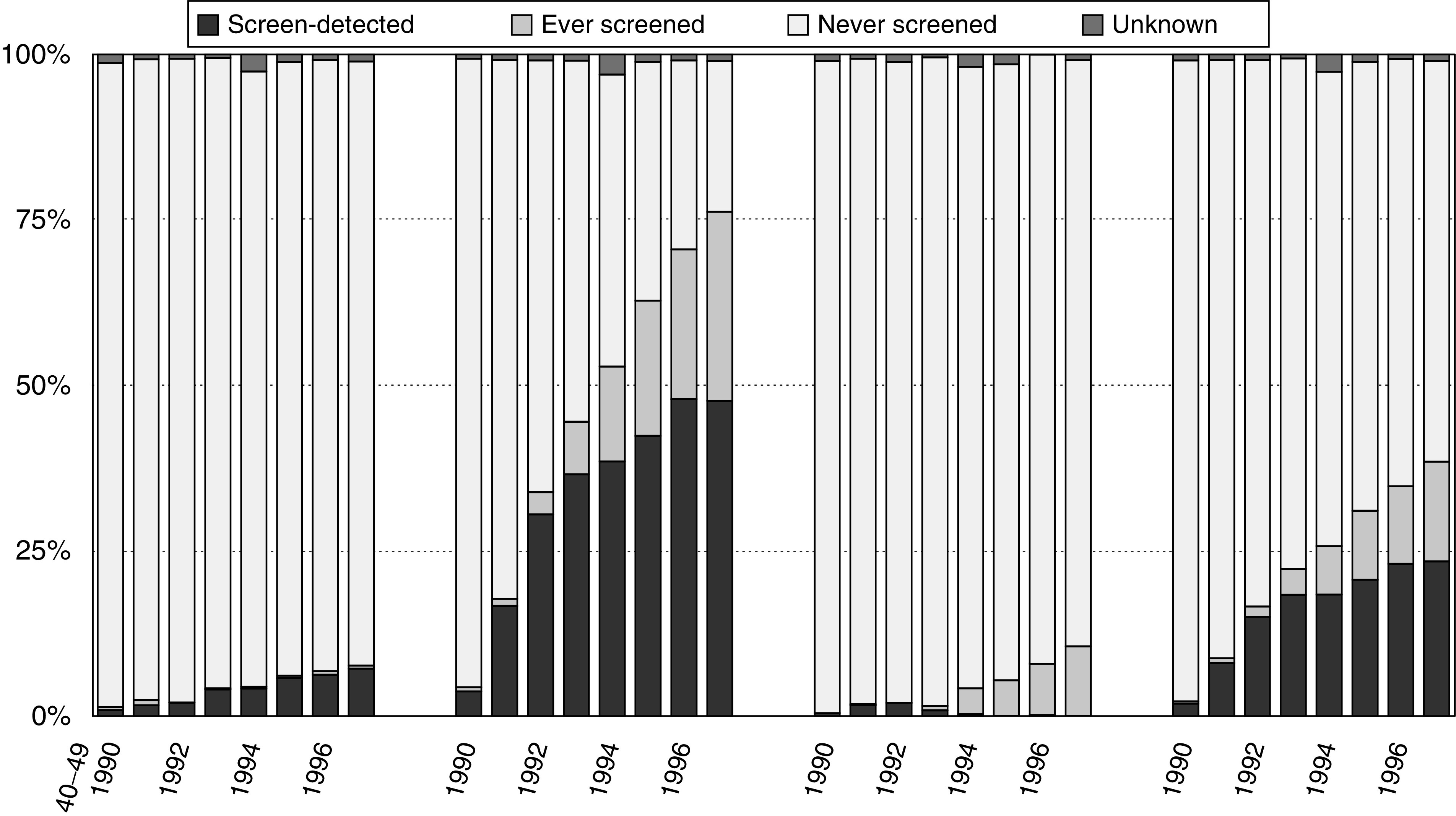
Percent distribution of newly diagnosed invasive and *in situ* breast cancers as related to screening (screen-detected; in ever-screened women, in never-screened women and unknown) by age category 1990–1997.

). In women aged 50–69 years, half of all breast cancers were screen-detected cancers and a quarter were symptomatic cancers in ever-screened women.

Figure 3

**Figure 3:**
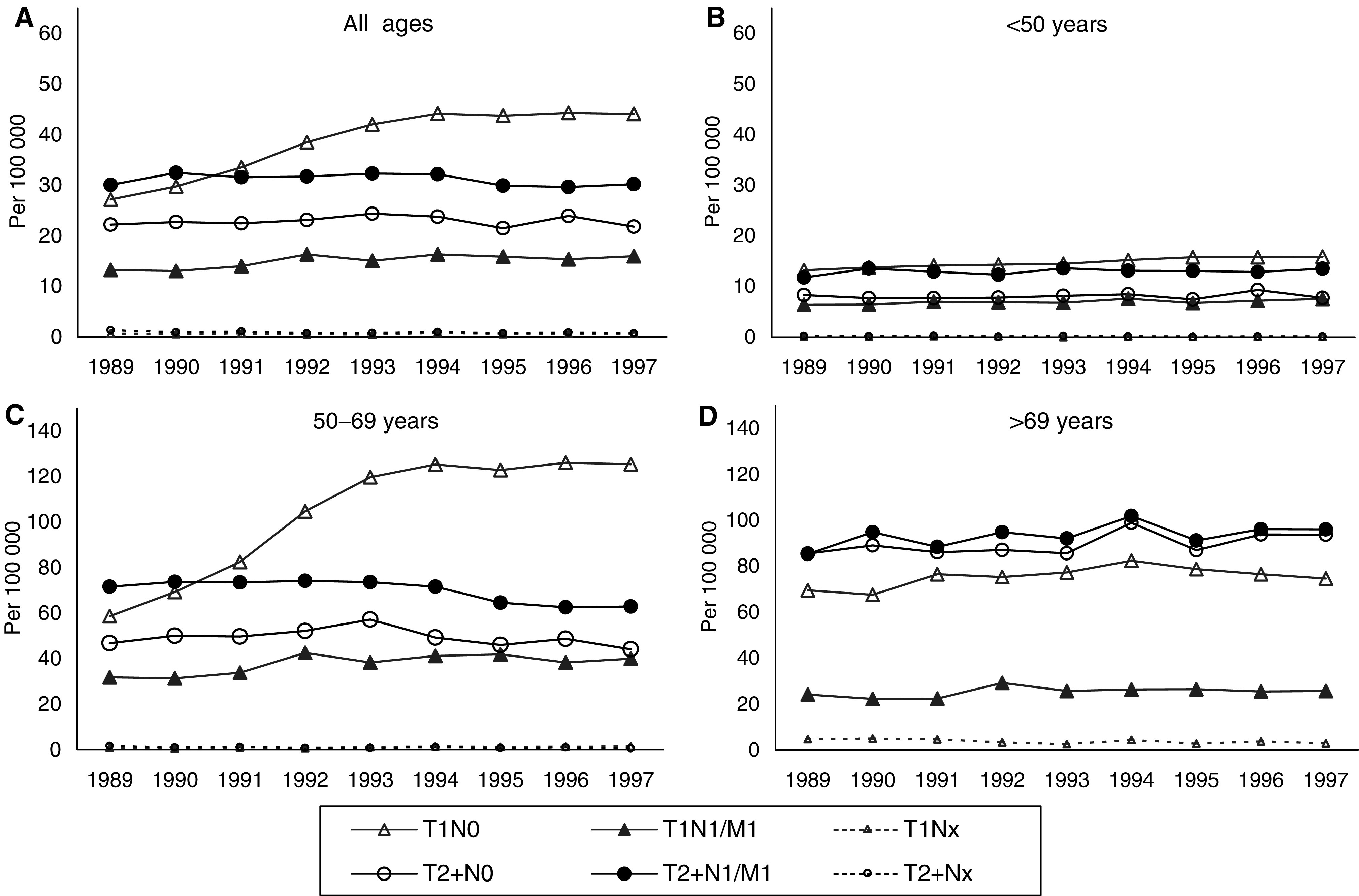
Age-adjusted invasive breast cancer rates (ESR) in 1989–1997 per 100 000 by tumour stage in seven ‘new’ regions: (**A**) all ages; (**B**) <50 years; (**C**) 50–69 years; (**D**) >69 years.

shows that the increase in overall invasive breast cancer rates (Figure 3a) was mainly caused by an increase of T1 tumours, especially lymph node negative T1 tumours (T1N0), and particularly in the age category 50–69 (Figure 3c). In this age category, the doubling of small lymph node negative tumours was followed by a decline in T2+ cancers with lymph node or distant metastases (T2+N+/M1). In younger and older women, the incidence rates of these cancers showed a nonsignificant slightly increasing trend (EAPC 0.84 and 1.09% for women aged <50 and >69 years, respectively; Table 2

**Table 2:** Annual age-adjusted incidence rates (ESR) and rate difference compared with 1989 of advanced breast cancers (T2+/N+/M1) per 100 000 women in the seven ‘new’ regions

	**T2+/N+/M1**			
	***N***	**Per 100 000**	**Rate difference compared to 1989**	**(95% CI)**
<50 years				
1989	496	11.8	—	
1990	584	13.6	1.8	(0.3, 3.3)
1991	569	12.9	1.2	(−0.3, 2.7)
1992	559	12.3	0.6	(−0.9, 2.0)
1993	633	13.7	1.9	(0.4, 3.4)
1994	619	13.1	1.4	(−0.1, 2.8)
1995	630	13.1	1.4	(−0.1, 2.8)
1996	621	12.9	1.2	(−0.3, 2.6)
1997	649	13.6	1.8	(0.3, 3.3)
EAPC			0.84	(−0.59, 2.29)
				
*50–69 years*				
1989	884	71.6	—	
1990	917	73.7	2.1	(−4.7, 8.9)
1991	914	73.6	2.0	(−4.8, 8.8)
1992	926	74.2	2.6	(−4.2, 9.4)
1993	920	73.7	2.1	(−4.7, 8.8)
1994	901	71.6	−0.0	(−6.7, 6.7)
1995	815	64.6	−7.0	(−13.6, 0.5)
1996	808	62.6	−9.1	(−15.5, −2.6)
1997	833	63.0	−8.6	(−15.1, −2.2)
EAPC			−2.14	(−3.47, −0.80)
				
>*69 years*				
1989	572	85.3	—	
1990	641	94.9	9.6	(0.3, 18.9)
1991	606	88.5	3.2	(−14.4, 20.8)
1992	664	94.9	9.6	(0.3, 18.8)
1993	661	92.1	6.8	(−2.3, 15.8)
1994	737	102.0	16.7	(7.3, 26.0)
1995	670	91.2	5.9	(−3.1, 14.9)
1996	726	96.3	11.0	(1.9, 20.0)
1997	734	96.1	10.8	(1.8, 19.8)
EAPC			1.09	(−0.31, 2.52)

EAPC: estimated annual percentage change.

). In women aged 50–69, after a moderate increase by approx. 3% up to 1994, incidence rates of advanced disease are significantly lower from 1996 onwards (EAPC −2.14; 95% CI −3.47, −0.80; Table 2). This resulted in a decrease by 12.1% in 1997 compared to that in 1989 (63.0 *vs* 71.6 per 100 000). In the ‘old’ regions, the decline in this age category was larger, with an EAPC of −5.5 (95% CI −8.52, −2.37; data not shown).

Table 3

**Table 3:** Annual age-adjusted rates (ESR) of advanced breast cancers (T2+/N+/M1) and breast cancer mortality in the Netherlands (all regions), and rate difference compared with observed rates in 1989 (advanced cancers) and in 1986–88 (mortality), women aged 55–74 years

	**Advanced cancers (T2+N+/M1)**			**Mortality^a^**		
	**Per 100 000**	**Rate difference**	**(95% CI)**	**Per 100 000**	**Rate difference**	**(95% CI)**
1986–88				105.2	—	
1989	93.6	—	—	102.9	−2.3	(−8.6, 4.0)
1990	95.6	2.0	(−6.1, 10.1)	107.5	2.3	(−4.1, 8.7)
1991	94.4	0.8	(−7.2, 8.9)	105.0	−0.2	(−6.6, 6.1)
1992	97.6	4.0	(−4.1, 12.2)	102.1	−3.2	(−9.4, 3.1)
1993	91.5	−2.0	(−10.0, 6.0)	101.1	−4.1	(−10.3, 2.1)
1994	92.0	−1.5	(−9.5, 6.4)	103.9	−1.3	(−7.6, 4.9)
1995	80.4	−13.1	(−20.8, −5.4)	100.2	−5.0	(−11.1, 1.2)
1996	80.4	−13.2	(−20.9, −5.4)	100.0	−5.2	(−11.4, 1.0)
1997	78.0	−15.6	(−23.2, −8.0)	98.2	−7.0	(−13.1, −0.9))
1998				92.1	−13.1	(−19.0, −7.1)
1999				97.8	−7.4	(−13.4, −1.3)
2000				89.7	−15.5	(−21.4, −9.7)
2001				85.3	−19.9	(−26.6, −14.2)

a Otto *et al* (2003).

compares the incidence rates of advanced breast cancers with the breast cancer mortality in The Netherlands (all nine regions, women aged 55–74) during the 1990s (Otto *et al*, 2003). This reduction of advanced diseases precedes the observed significant breast cancer mortality reduction of a comparable extent by approximately 2 years.

## DISCUSSION

The implementation of the nation-wide breast cancer screening programme in The Netherlands from 1990 to 1997 coincided with an obvious increase in the incidence rates of both invasive and *in situ* breast cancers. This increase was strongest in women aged 50–69 years, the main target population for the programme, especially in the early 1990s, when predominantly initial screen examinations were carried out. The average detection rate was at that time 6.1 per 1000 initially screened women (NETB (National Evaluation Team for Breast cancer screening in The Netherlands), 2001). In 1995–1997, with a large majority of subsequent screen examinations (average detection rate 3.4 per 1000 screened women), the overall incidence fell to a lower level. It remained, however, above the 1989 baseline. This trend is consistent with the expected development of the breast cancer incidence that had been predicted previously by our group (de Koning *et al*, 1990). We found an estimated annual percentage change (EAPC) of 2.46 with respect to the overall incidence including ductal carcinoma *in situ*. In their last report, the NCR calculated for invasive breast cancers only an EAPC of 1.5% (*P*-value 0.0009) for the period 1989–2000 (Visser *et al*, 2003).

The proportion of breast cancers that were diagnosed as a result of a screen examination increased gradually during the study period. In 1997, when the screening programme was fully implemented in The Netherlands, one out of four breast cancers was screen-detected and this ratio was even one out of two in women aged 50–69 years. In this age category, another 25% of the breast cancers were diagnosed during the screening interval after a negative screen examination. The remaining 25% were diagnosed in nonscreened women.

Various studies described an increase in breast cancer incidence rates in the last two to three decades for both, situations with and without organised mammography screening (Quinn and Allen, 1995; Chu *et al*, 1996; McCann *et al*, 1998; Kricker *et al*, 1999; Paci *et al*, 2002; Schouten *et al*, 2002; Botha *et al*, 2003; Buiatti *et al*, 2003). Where organised screening has been introduced, this increase is more marked, mainly as a result of the additional detection of early breast cancer stages. The extent of the increase is – besides the country-specific underlying incidence – directly related to the intensity of the screening programme, for example, the targeted proportion of the female population, the speed of implementation, the screening interval, the quality of the programme and the participation rate (Threlfall *et al*, 2003). In most studies, a decline was observed after the initial screening round had been finished. Those studies with a sufficiently long follow-up provided valuable insight into the course of the total breast cancer incidence during subsequent screens. In general, the incidence remains higher than in the prescreening period, which raises the question whether the ongoing subsequent screening leads to overdiagnosis or whether the breast cancer risk is increasing. It is important to realise that the initial increase does not signify overdiagnosis, but that it is the result of the necessary downstaging of breast cancer diagnoses, if screening will be effective. Several authors concluded that overdiagnosis might be limited to a few percent (Boer *et al*, 1994; Olsen *et al*, 2003; Yen *et al*, 2003; Paci *et al*, 2004). It is likely that besides organised screening and the general tendency towards earlier detection (increased awareness, spontaneous mammography) other factors, such as declining fertility rates and the widespread use of hormone replacement therapy, contribute to the observed increase in breast cancer incidence (Prehn *et al*, 2002; Beral, 2003; Coebergh, 2003; Li *et al*, 2003).

The present study confirms that relatively more small lymph node negative cancers were detected in the targeted age category 50–69 after the start of the programme. More importantly, in this same age group, the incidence rate of large cancers with lymph node or distant metastases began to decline significantly in 1996. In 1997, this rate was a significant 12.1% lower than that in 1989. We defined advanced breast cancer as tumour size T2+ in combination with positive lymph nodes and/or distant metastases. This does not fully correspond with the TNM stages IIB–IV, because we did not take into account node-negative T4 cancers (stage IIIB, in total 117–184 per year), T4 cancers with unknown lymph node findings (stage IIIB, 24–40 per year) and tumours TX with distant metastases (stage IV, 40–56 per year). Given the fact that we selected the worst cases of large tumours, the reduction of advanced cancers in women aged 50–69 is strongly indicative of a screening effect, especially since the other age categories showed no tendency towards lower incidence rates of these cancers. We found a similar pattern in the two ‘old’ regions, but the decrease in advanced cancers in women aged 50–69 was much more pronounced (EAPC −5.50; 95% CI −8.52, −2.37), resulting in a 38.7% lower rate in 1997 compared with 1989 (data not shown). If the ‘old’ regions, where the programme was already fully implemented in 1993–1994, predict the trend for the nation-wide programme, then we can expect a substantial further reduction in the advanced cancer incidence in the next years.

In the seven ‘new’ regions, the decline in advanced cancer rates in women aged 50–69 began in the 7th year of screening activities when approx. 85% of the targeted population had been invited at least once. Day *et al* (1989) suggested a rate reduction of advanced cancers of at least 30% 7 years after the first invitation is sent. Our results are far from this desirable level. One of the reasons is that the implementation of a nation-wide programme takes much longer than that of a smaller screening trial, in the Dutch situation, even more than 7 years. Differences in screening performance may be another reason. As far as we know, the published reductions of advanced cancer rates in regions with a service screening programme have not reached 30% up to now. New South Wales reported a 20% reduction of large (T3+) cancers in women aged 50–69 years (Kricker *et al*, 1999). For the East Anglia region, the estimated reduction of advanced stages II–IV was 7–19% after 7 years (McCann *et al*, 1998). In the small Dutch region Limburg, one of the seven ‘new’ regions, the incidence of stage II–IV cancers was reduced by an estimated 10% and 18% after 5 and 10 years (Schouten *et al*, 1998, 2002). Based on NCR data, a 24% reduction was reported for the combined stages III and IV in 1995 (van Dijck *et al*, 2000). Our results are largely in line with these outcomes, despite the somewhat different definition of advanced disease.

Assuming that women with advanced cancers will mostly die of breast cancer, the reduction in advanced disease by screening can be regarded as a predictor of the future breast cancer mortality reduction. In fact, the breast cancer mortality in Dutch women aged 55–74 started to decline significantly from 1997 onwards (Otto *et al*, 2003). After recalculation of the incidence rates for the same age band, we found a significant reduction of advanced diseases already in 1995, preceding the breast cancer mortality reduction of a similar extent by 2 years (Table 3).

## CONCLUSION

Breast cancer screening led to a temporary strong increase in the breast cancer incidence, in particular of *in situ* carcinomas and small lymph node negative invasive cancers in women aged 50–69 years. In this age category, the incidence rate of large tumours with lymph node or distant metastases (T2+N+/M1) decreased significantly and was 12.1% lower in 1997 than before the start of the screening programme. This reduction in advanced disease precedes the observed significant breast cancer mortality reduction of a comparable extent by approximately 2 years. The outcomes of our study confirm that population-based mammography screening contributes to a reduction of advanced breast cancer rates and breast cancer mortality.
